# Standardization and diagnostic utility of the Frontal Assessment Battery for healthy people and patients with dementia in the Chilean population

**DOI:** 10.1590/1980-5764-DN-2021-0059

**Published:** 2022

**Authors:** Fabrissio Grandi, David Martínez-Pernía, Mario Parra, Loreto Olavarria, David Huepe, Patricia Alegria, Álvaro Aliaga, Patricia Lillo, Carolina Delgado, Marcela Tenorio, Ricardo Rosas, Oscar López, James Becker, Andrea Slachevsky

**Affiliations:** 1 Gerosciences Center for Brain Health and Metabolism, Santiago, Chile.; 2 Universidad de Chile, Faculty of Medicine, Hospital del Salvador, Memory and Neuropsychiatric Clinic, Neurology Department, Santiago, Chile.; 3 Universidad de Chile, Faculty of Medicine, Neuropsychology and Clinical Neuroscience Laboratory, Physiopathology Department, Neuroscience and East Neuroscience Departments, Santiago, Chile.; 4 Universidad de los Andes, School of Psychology, Santiago, Chile.; 5 Universidad Adolfo Ibañez, School of Psychology, Center for Social and Cognitive Neuroscience, Santiago, Chile.; 6 University of Strathclyde, School of Psychological Sciences and Health, Glasgow, Scotland.; 7 Clínica Alemana, Physical Medicine and Rehabilitation Service, Santiago, Chile.; 8 Diego Portales Universidad, School of Psychology, Santiago, Chile.; 9 Universidad de Chile, Faculty of Medicine, South Neuroscience Department, Santiago, Chile.; 10 Complejo Hospitalario San José, Neurology Unit, Santiago, Chile.; 11 Universidad de Chile, School of Medicine, Department of Neuroscience, Santiago, Chile.; 12 Universidad de Chile, Hospital Clínico, Department of Neurology and Neurosurgery, Healthy Brain Unit, Santiago, Chile.; 13 Millennium Institute for Caregiving Research, Santiago, Chile.; 14 Pontificia Universidad Católica de Chile, Center for the Development of Inclusion Technologies, Santiago, Chile.; 15 University of Pittsburgh, Department of Psychiatry, Pittsburgh, PA, USA.; 16 University of Pittsburgh, Department of Neurology, Pittsburgh, PA, USA.; 17 University of Pittsburgh, Department of Psychology, Pittsburgh, PA, USA.; 18 Universidad del Desarrollo, Clínica Alemana, Department of Medicine, Neurology Unit, Santiago, Chile.

**Keywords:** Executive Function, Dementia, Mental Status and Dementia Tests, Neurodegenerative Diseases, Função Executiva, Demência, Testes de Estado Mental e Demência, Doenças Neurodegenerativas

## Abstract

The Frontal Assessment Battery (FAB) is a screening test that measures executive functions. Although this instrument has been validated in several countries, its diagnostic utility in a Chilean population has not been studied yet.

**Objectives:**

This study aimed to (1) adapt FAB in a Chilean population; (2) study the psychometric properties of the FAB in a Chilean population; (3) assess the sociodemographic influence in the performance of the FAB in a sample of healthy controls (HC); and (4) develop normative data for this healthy group.

**Methods:**

A HC (n=344) and a group of patients with dementia (n=156) were assessed with the Chilean version of FAB.

**Results:**

FAB showed good internal consistency (Cronbach's alpha=0.79) and acceptable validity based on the relationship with other variables. Factor analysis showed the unidimensionality of the instrument. Significant differences were found in the total FAB value between the HC and dementia groups. With the matched sample, the established cutoff point was 13.5, showing a sensitivity of 80.8% and a specificity of 90.4%. Regression analysis showed that education and age significantly predicted FAB performance in the healthy group. Finally, normative data are provided.

**Conclusions:**

This study shows that FAB is a useful tool to discriminate between healthy people and people with dementia. However, further studies are needed to explore the capacity of the instrument to characterize the dysexecutive syndrome in people with dementia in the Chilean population.

## RESUMO.

A Bateria de Avaliação Frontal (FAB) é um teste de rastreio que mede as funções executivas. Embora esse instrumento tenha sido validado em vários países, sua utilidade diagnóstica em uma população chilena ainda não foi estudada.

**Objetivos:**

(1) Adaptar a FAB para uma população chilena; (2) estudar as propriedades psicométricas da FAB em uma população chilena; (3) avaliar a influência sociodemográfica no desempenho da FAB em uma amostra de controles saudáveis; e (4) desenvolver dados normativos para este último grupo.

**Métodos:**

Um grupo controle saudável (n=344) e um grupo de pacientes com demência (n=156) foram avaliados com a versão chilena da FAB.

**Resultados:**

A FAB apresentou boa consistência interna (alfa de Cronbach=0,79) e validade aceitável com base na relação com outras variáveis. A análise fatorial mostrou a unidimensionalidade do instrumento. Diferenças significativas foram encontradas no valor total da FAB entre os grupos controle saudável e demência. Com a amostra pareada, o ponto de corte estabelecido foi de 13,5, que apresentou sensibilidade de 80,8% e especificidade de 90,4%. A análise de regressão mostrou que a escolaridade e a idade predisseram significativamente o desempenho da FAB no grupo saudável. Finalmente, os dados normativos são fornecidos.

**Conclusões:**

O presente estudo mostrou que a FAB é uma ferramenta útil para discriminar entre pessoas saudáveis e aquelas com demência. No entanto, mais estudos são necessários para explorar a capacidade do instrumento para caracterizar a síndrome disexecutiva em pessoas com demência na população chilena.

## INTRODUCTION

The executive function (EF) comprises a wide range of cognitive processes and behavioral competencies, including reasoning, problem-solving, planning, sequencing, resistance to interference, multitasking, cognitive flexibility, and the capacity to deal with novelty, among others^1^. These processes mainly depend on neural circuits involving the prefrontal cortex, the basal ganglia, the parietal cortex, the cerebellum, and the thalamus^2^. Assessing EF can be helpful in the diagnosis and prognosis of many brain disorders and other neuropsychiatric conditions, such as vascular cognitive impairment, frontotemporal dementia, parkinsonian disorders, and schizophrenia^3^. Along with the comprehensive neuropsychological evaluation of executive dysfunction, brief screening tools that are easy and quick to administer and contribute to determining whether a person presents with executive impairments and, accordingly, improving the quality of preliminary diagnostic workup are used^4,5^. In this context, the Frontal Assessment Battery (FAB) was devised as a rapid bedside screening of frontal functions. The FAB comprises six subtests that assess different domains of EF^5^. Each subset explores a specific cognitive or behavioral domain related to the functions of frontal lobes, including conceptualization, mental flexibility, motor programming, sensitivity to interference, inhibitory control, and environmental autonomy. The global performance on these six subtests gives a composite score that summarizes the severity of the dysexecutive syndrome^6^. The FAB has good correlations with other executive measures such as the Wisconsin Card Sorting Test (WCST) (number of perseverative errors: rho=0.68; and number of criteria: rho=0.77) as well as measures of general cognitive functioning (Mattis Dementia Rating Scale) (rho=0.82)^5^.

Since its first publication, the FAB has been adapted to diverse languages and cultures, including Brazil^7^, Korea^8^, Japan^9^, Italy^10^, Germany^11^, France^5,12^, China^13^, Portugal^14^, Spain^15^, Turkey^16^, Taiwan^17^, and Persia^18^. Several studies have reported that the FAB has presented adequate reliability and validity.

The diagnostic utility of the FAB has been reported in patients with Alzheimer's disease^8^, amyotrophic lateral sclerosis^19^, frontotemporal dementia^12^, and in small study of patients with stroke^13^. Age, education, and race influence the performance in executive tests^16,20,21^. Although some empirical work has been done on FAB in Latin America^7,21^, there has not been yet any studies in this region that provide normative data in Spanish. More studies are needed in Spanish-speaking Latin America and the Caribbean (LAC) countries to support its use in clinical practice.

Therefore, our aims were to (a) adapt FAB in a Chilean population; (b) study the psychometric properties of the FAB in this population (healthy people and people with dementia); (c) assess the influence of sociodemographic variables in the performance of the FAB in the healthy controls (HC); and (d) develop normative data in this healthy group.

## METHODS

### Participants

This normative study involved 344 HC (194 women and 150 men). All of them were native Spanish speakers (Chilean), lived in the community, and met the following inclusion criteria: (a) with at least a minimal writing capacity (correct writing regardless of orthographic errors due to low education); (b) scores >24/30 on the Mini-Mental State Examination (MMSE)^20^ (c); scores <5 on the Geriatric Depression Scale^22^ (d); scores <51 in the Zung Anxiety Scale^23^. Subjects were excluded if they had current major psychiatric diseases including alcohol or drug abuse, were taking psychoactive drugs, had history of brain injury (e.g., stroke, dementia, or any other neurological illness detected on a semi-structured clinical interview), or had a severe sensory deficit (loss of vision and/or hearing) that could impede neuropsychological evaluation.

They were recruited through a variety of advertisements at citizen activity centers and workplaces. Participation was voluntary, and the participants did not receive any compensation for their contribution to the study. This study was approved by the Comité de Ética of the Servicio Metropolitano Oriente, Santiago, Chile. Written informed consent was obtained from all the participants.

The clinical sample included 156 patients with dementia syndromes (83 women and 73 men) (Table 1). All patients were evaluated in the Cognitive Neurology and Dementia Unit (UNCD) at the Department of Neurology, Hospital del Salvador in Santiago, Chile. The UNCD receives patients with suspected dementia from primary care facilities. A diagnosis was made by a neurologist based on the *DSM-IV-TR* criteria for dementia using multidisciplinary approach (neurological, neuropsychological, laboratory, and neuroimaging data). There were 115 patients with Alzheimer's disease, 17 with frontotemporal dementia behavioral variant, 6 with Lewy body dementia, 3 with vascular dementia, 2 with mixed dementia, 1 with semantic dementia, 1 with progressive supranuclear Palsy, 1 with alcoholic dementia, and 10 with dementia of unknown etiology.

**Table 1:** Demographic and neuropsychological characteristics of healthy controls and patients.

	Control (n=344)	Dementia (n=156)	p-value
Age^a^	55.35±18.096	74.2±7.626	<0.001
Years of education^a^	12.62±4.565	11.42±4.7	<0.01
MMSE^a^	28.53±1.578	19.68±5.631	<0.001
FAB total^a^	16.07±1.751	10.06±3.865	<0.01
FAB subtest 1^a^	2.32±0.685	1.5±1.002	<0.01
FAB subtest 2^a^	2.73±0.530	1.56±1.013	<0.01
FAB subtest 3^a^	2.53±0.755	1.42±0.993	<0.01
FAB subtest 4^a^	2.86±0.402	1.82±1.187	<0.01
FAB subtest 5^a^	2.63±0.729	1.26±1.133	<0.01
FAB subtest 6^a^	2.98±0.178	2.48±0.963	<0.01

a Results are expressed in mean±standard deviation;

MMSE: Mini-Mental State Examination; FAB: Frontal Assessment Battery.

### Instruments and procedure

All participants were initially assessed with the MMSE. The adaptation of the FAB to Spanish was achieved by two translations from English to Spanish based on the original FAB, followed by two back-translations from Spanish to English that were reviewed with one of the authors’ original FAB. The forward- and back-translations were performed independently by different individuals, in each case by one bilingual expert in the field of dementia and by one bilingual layperson. The Chilean version of the FAB (FAB-Ch) can be found in the Supplementary material (Appendix 1). It maintains the structure and number of items of the original English version and is grouped into six sections: conceptualization, mental flexibility, motor programming, sensitivity to interference, inhibitory control, and environmental autonomy. The original lexical fluency task with letter “S” in the English version was changed to lexical fluency with letter “A” because the number of words starting with A in Spanish is higher than those starting with letter S. Each subtest is scored from 3 (high score) to 0 (low score). The maximum score is 18 points.

### Statistical analyses

All analyses were conducted using IBM SPSS Statistics 25 for Microsoft Windows (IBM Corp., Armonk, NY, USA). Descriptive and comparative analyses were performed using Student's *t*-tests to compare between the two groups. Regarding psychometric aspects, reliability was explored via internal consistency of the instrument with Cronbach's alpha.

Evidence of validity based on the relationship with other variables was evaluated by assessing the association between the performance on the FAB-Ch and MMSE. We also studied the correlation between our instrument and two EFs tests: (a) number of sorts in WCST and (b) categorical fluency, collected in the Chilean-Argentine version of the ACE-III test^24^ using Pearson's correlation. In case of categorical fluency, the participant must identify the names of animals.

The diagnostic utility was determined using the Receiver Operating Characteristic (ROC) analysis to calculate sensitivity and specificity values. The first analysis was carried out with the complete sample, and the second analysis included the matched sample in the variables age and education. The influence of sociodemographic variables in the HC was also studied using linear regression. Finally, we present mean and standard deviation (SD) of the total FAB-Ch scores stratified by age and education, as well as scores in the single subtests of this instrument.

## RESULTS

### Sociodemographic variables

Demographic and neuropsychological data of the sample are presented in Table 1. The HC was younger [t(502)=-12.485, p<0.001] and showed more years of education [t(502)=-2.639, p<0.01] than the patients group. People with dementia performed significantly worse than the HC on the MMSE [analysis of covariance (ANCOVA) covaried by age and years of education: F(1,499)=579.60; p<0.001]. In the case of the HC, the proportion of women was 53.2% and that of men was 45.8%, while dementia patients showed a proportion of 56.4% for women and 43.6% for men. This last case is probably associated with epidemiological variables. Alternatively, to control the effect of demographic variables on the difference between HC and patients group, we performed an analysis in a subsample of participants matched by age and education level. The outcome was very similar to that obtained from total data (Table 2).

**Table 2:** Demographic and neuropsychological characteristics of healthy controls and patients matched by age and education level.

	Control (n=122)	Dementia (n=118)	p-value
Age^a^	72.25±6.891	70.84±6.73	0.11
Years of education^a^	11.63±4.857	12.61±4.861	0.117
MMSE^a^	28.29±1.639	20.06±5.307	<0.001
FAB total^a^	15.77±1.875	10.35±3.925	<0.001
FAB subtest 1^a^	2.36±0.739	1.57±0.977	<0.001
FAB subtest 2^a^	2.66±0.625	1.55±1.038	<0.001
FAB subtest 3^a^	2.43±0.862	1.5±0.988	<0.001
FAB subtest 4^a^	2.86±0.44	1.88±1.176	<0.001
FAB subtest 5^a^	2.42±0.87	1.28±1.136	<0.001
FAB subtest 6^a^	2.99±0.091	2.54±0.905	<0.001

a Results are expressed in mean±standard deviation;

MMSE: Mini-Mental State Examination; FAB: Frontal Assessment Battery.

### Performance on Chilean version of the Frontal Assessment Battery: total score and subtests

Significant differences were found in the total FAB-Ch values between the HC and the dementia group (Table 1). Regarding the scores obtained in the subtests that make up the FAB-Ch, significant differences were again found between the two study groups in the domains of “conceptualization,” “mental flexibility,” “motor programming,” “sensitivity to interference,” “inhibitory control,” and “environmental autonomy.” Additionally, a multivariate analysis of covariance (MANCOVA) was conducted to compare results across subtests of the FAB-Ch by diagnosis category controlling for age, sex, and years of education. Performance differed significantly between the two groups for each subtest of the FAB-Ch [Wilks’ lambda=0.488, F(6,487)=77.352; p<0.001]. Both HC and patients group differed in each of the subtests: Conceptualization [F(1,492)=91.176, p<0.001]; mental flexibility [F(1,492)=198.732, p<0.001]; motor programming [F(1,492)=141.212, p<0.001]; sensitivity to interference [F(1,492)=171.490, p*<*0.001]; inhibitory control [F(1,492)=148.245, p*<*0.001]; and environmental autonomy [F(1,492)=60.176, p<0.001].

### Psychometric properties

#### Reliability

The Cronbach's alpha for the FAB-Ch considering the 6 subscales and calculated for all 500 subjects was α=0.797, which shows a good reliability of the instrument. Cronbach's values of the six subtests suggest that all items positively contributed to the overall reliability.

#### Validity based on the relationship with other variables

The FAB-Ch showed a statistically significant association with the MMSE (Pearson's r=0.83; p<0.001, n=499) and other measures of EF (number of sorts in WCST: r=0.678, p<0.001, n=413; and category fluency: r=0.71, p<0.001, n=493) collected in the ACE-III test, so we have a high validity based on the relationship with other variables^25^.

#### Structure of the Chilean version of the Frontal Assessment Battery

The six subscales of the FAB-Ch were subjected to an Exploratory Factorial Analysis in order to obtain its factorial structure. We used Kaiser's criterion (eigenvalues>1.0) and the extraction method was by principal axis factoring. The factors were then orthogonally rotated using a varimax rotation. The Kaiser–Meyer–Olkin test for sampling adequacy was 0.85, which indicates that factor analysis is appropriate. Bartlett's test of sphericity reached statistical significance (χ^2^=805.95, p<0.001), supporting the factorability of the correlation matrix. The results showed that the FAB-Ch has a unidimensional structure. The explained variance was 41%, and the factorial loadings were mostly above 0.5.

#### Utility of the Chilean version of the Frontal Assessment Batteryto classify patient and healthy controls

**Figure 1:**
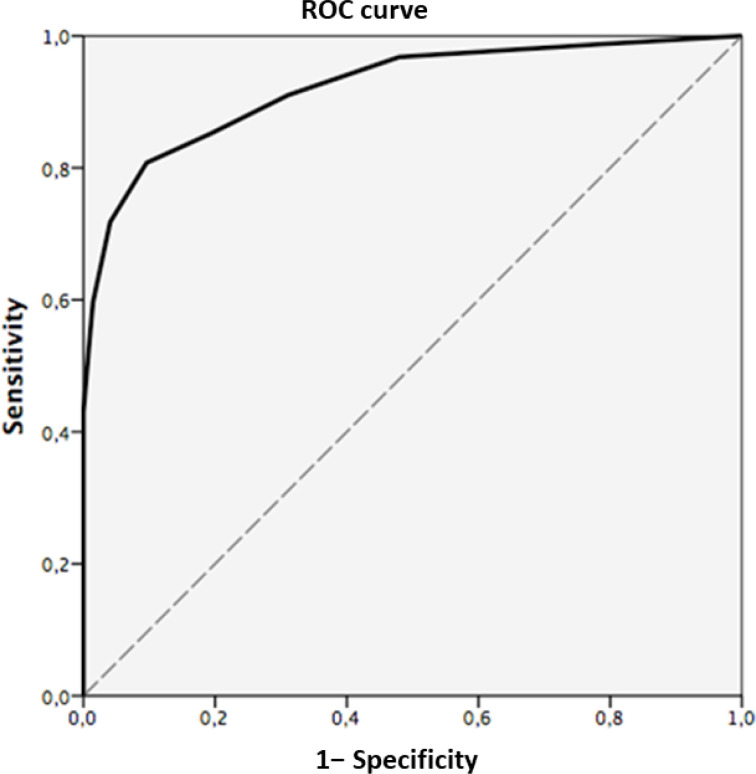
Receiver Operating Characteristic curve for Frontal Assessment Battery score in healthy controls and dementia patients.

The results of the ROC curve analysis for the FAB are shown in Table 3 and Figure 1. The area under the curve (AUC) for the FAB was 0.92 (95% confidence interval: 0.89–0.95), indicating an overall high diagnostic usefulness of the test^26^. The optimal balance between sensitivity and specificity for the FAB was obtained with a cutoff point of 13.5 (sensitivity=80.8%, specificity=90.4%).

**Table 3:** Sensitivity and specificity of the Chilean version of the Frontal Assessment Battery for the discrimination of dementia patients (n=156) and healthy controls (n=344) in the complete sample.

Cutoff point	Sensitivity	Specificity
-1.00	0.000	1.000
1.00	0.006	1.000
2.50	0.026	1.000
3.50	0.038	1.000
4.50	0.064	1.000
5.50	0.141	1.000
6.50	0.212	1.000
7.50	0.282	1.000
8.50	0.359	1.000
9.50	0.429	1.000
10.50	0.532	0.991
11.50	0.596	0.985
12.50	0.718	0.959
13.50	0.808	0.904
14.50	0.853	0.805
15.50	0.910	0.689
16.50	0.968	0.520
17.50	0.987	0.218
19.00	1.000	0.000

**Figure 2:**
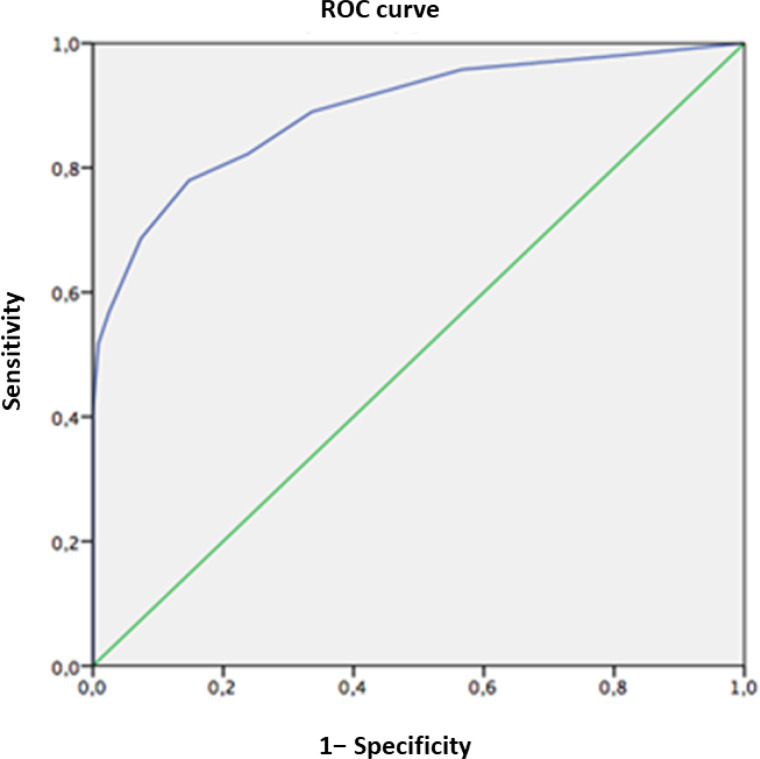
Receiver Operating Characteristic curve for Frontal Assessment Battery score in healthy controls and dementia patients matched by age and education level.

Finally, we evaluated the sample matched by age and education level. The optimal balance between sensitivity and specificity for the FAB was again obtained with a cutoff point of 13.5 (Table 4 and Figure 2).

**Table 4:** Sensitivity and specificity of the Chilean version of the Frontal Assessment Battery on healthy controls (n=122) and patients (n=128) matched by age and education level.

Cutoff point	Sensitivity	Specificity
-1.00	0.000	1.000
1.00	0.008	1.000
2.50	0.025	1.000
3.50	0.042	1.000
4.50	0.051	1.000
5.50	0.110	1.000
6.50	0.195	1.000
7.50	0.263	1.000
8.50	0.339	1.000
9.50	0.407	1.000
10.50	0.517	0.992
11.50	0.568	0.975
12.50	0.686	0.926
13.50	0.780	0.852
14.50	0.822	0.762
15.50	0.890	0.664
16.50	0.958	0.434
17.50	0.983	0.164
19.00	1.000	0.000

### Influence of sociodemographic variables in the healthy controls

Multiple regression analysis was used to test whether sociodemographic variables (i.e., gender, age, and years of education) significantly predicted FAB-Ch performance in the normative sample. The results of the regression indicated these predictors explained 34.9% of the variance [r^2^=0.349, F(3,344)=60.796, p<0.001]. Both education [β=0.569, t(344)=12.831, p<0.01] and age [β=-0.127, t(344)=-0.127, p<0.01] significantly predicted FAB-Ch score. Based on this analysis, we calculated an FAB-Ch predicted value for each patient using the formula: 13.977−0.012×age (years)+0.218×education (years). We then subtracted the patient's actual score on the FAB-Ch score from the predicted score. The mean difference between FAB-Ch observed score (10.06±3.86) and the FAB-Ch predicted score (15.79±1.03) was −5.73 (SD=3.67). This value is significantly different from zero [t(155)=-19.53, p<0.001].

### Normative data in the healthy control group

We created a table of normative values based only on age and education. Table 5 shows the normative data for total scores for the FAB-Ch in the HC group.

**Table 5:** Normative data for the Frontal Assessment Battery score total.

Age	n	Education	Mean	SD	Median	Maximum	Minimum
20–49	30	0–8	15.00	1.23	15.00	17.00	13.00
	29	9–12	15.86	1.83	16.00	18.00	10.00
	66	13 or +	17.11	1.14	17.00	18.00	13.00
50–69	30	0–8	14.17	1.76	14.00	17.00	10.00
	25	9–12	16.04	1.67	16.00	18.00	12.00
	63	13 or +	16.79	1.25	17.00	18.00	13.00
70–89	26	0–8	14.00	1.72	14.00	17.00	10.00
	22	9–12	16.14	1.32	16.00	18.00	13.00
	53	13 or +	16.74	1.26	17.00	18.00	12.00

SD: standard deviation.

## DISCUSSION

This report describes the standardization of the FAB-Ch in a Chilean sample of an HC and patients with dementia syndrome. We provide psychometric evidence and normative data of this instrument.

Regarding psychometric properties, the FAB-Ch has strong evidence of reliability based on internal consistency, similar to the data reported in previous studies^5,18^. The Chilean version has a high correlation with two measures of EFs: the WCST and categorical fluency, which provides an acceptable validity based on the relationship with other variables. However, it is important to highlight that categorical fluency is not a pure executive test and depends also on language and semantic memory^27–29^. Nevertheless, Junquera et al.^30^ showed that the executive component of this instrument significantly predicted conversion to dementia (1 year later) in patients with mild cognitive impairment who presented a dysexecutive phenotype, independently of impairment at baseline. This result is consistent with other studies with different populations showing that the FAB-Ch has appropriate convergent validity for testing frontal lobe function^13,17^.

We also found that the FAB-Ch strongly correlated with the MMSE, which is a measure of global cognitive function, which is different from previous results^5,13,31,32^. These results are unexpected since the MMSE does not formally evaluate EFs^33^. One possible explanation for this finding is the interaction between education and MMSE performance, with the former being associated with the FAB-Ch^13^. An alternative explanation is that FAB is sensitive to the disease progression, making it useful to monitor the clinical course of dementing diseases.

The factor analysis identified a single factor explaining most of the variance of the FAB-Ch, similar to previous findings^34^. The optimal balance between sensitivity and specificity for the FAB-Ch was obtained with a cutoff point of 13.5, highlighting that this test can discriminate between HC and people with dementia syndrome.

Performance on the FAB-Ch is explained by education and age, while gender does not contribute to performance. Cognitive aging is associated with a mild decline in EF^35,36^, and education affects performance on executive tests^37,38^. Our results are consistent with previous data on the effect of sociodemographic factors on the FAB-Ch^7,8,10,11,34,39,40^.

The availability of a normative sample including people with a wide range of educational levels is essential for using FAB-Ch in clinical practice, especially in countries like Chile, where the range of educational levels in the populations is very heterogeneous wide^8^.

Several limitations warrant consideration in generalizing our observations. First, although we have participants of different ages and educational ranges, the variability of the data is small, which could impact on the relative position of an individual concerning standard scores. Therefore, assessors interpreting FAB-Ch scores should always review the overall distribution of scores on this test and consider the raw score obtained by the individual, which could be especially important when, for example, trying to determine if a person's score is far outside the normal range^41^. Second, the main limitation of our study is that we only provide indirect evidence of the ability of the FAB-Ch to detect a dysexecutive syndrome (validity based on the relationship with other variables). We did not provide specific evidence of the utility of the FAB-Ch in the diagnosis of a dysexecutive syndrome. In this way, it is important to note that as has been highlighted for other screening instruments, FAB-Ch cannot lead to the specific diagnosis of the type of dementia, such as Alzheimer's disease or frontotemporal dementia^4^. The aim of FAB-Ch is to establish the presence and degree of severity in a specific domain (not the type of diagnosis)^4^. Emphasizing this limitation is particularly important since executive dysfunction is present in many dementia syndromes (e.g., Lewy body dementia, vascular dementia, frontotemporal dementia, and Alzheimer's disease)^42–44^.

In this study, we did not consider types of dementia in the analysis as its aim was to investigate the sensitivity and specificity of this screening tool relative to FC. In this line, we do not have measures of the level of severity of dementia from the point of view of functionality or a global level of severity of dementia such as the Global Deterioration Scale (GDS). However, we have the MMSE, a cognitive screening test that is widely used as a measure of cognitive severity, which can reduce this limitation

In addition, illiterate subjects were excluded, and only 13 participants of the HC have 4 years of education or below. Therefore, our norms have limited use for people with low educational level, who are still an important percentage of the Latin American population^45^. More studies are needed to establish norms in subjects with a low socioeconomic status. This study included only Chilean subjects, consequently limiting our data to other Spanish-speaking countries. Yet, recent normative data for 10 Spanish-language neuropsychological tests in 11 Latin American countries suggest that most of the differences in test performance are explained by age and educational factors. Inter-country factors only account for a small proportion of variance^46^. Finally, the inclusion of HC whose performances are 24 or higher on the MMSE could be criticized as too strict. However, as 95.9% of our sample has more than 4 years of education, this criterion ensured the inclusion of healthy subjects without cognitive impairment^47^.

In conclusion, the main results of our study are (a) the FAB-Ch is an instrument with strong evidence of reliability and validity based on international standard, (b) an adequate diagnosis utility for dementia, (c) the effect of aging and level of education on FAB-Ch performances, and (d) the availability of normative data for the FAB-Ch, improving the usefulness of this instrument in clinical settings. In addition to other tests such as the MMSE, the administration of the FAB-Ch allows a more comprehensive evaluation in the diagnosis process of dementia. Future studies need to address if FAB-Ch presents good diagnostic utility to show the degree of executive dysfunction and its contribution in the differential diagnosis of types dementia.

## APPENDIX 1 CHILEAN’S VERSION OF THE FAB

### 1. Semejanzas (conceptualización).

*“¿En qué se parecen…?”*

- *Un plátano y una naranja.*
- *Una mesa y una silla*
- *Un tulipán, una rosa y una margarita.*

Ayudar al paciente en caso de fracaso total “no se parecen” o parcial “los 2 tienen cáscara” en el primer ítem, no en los siguientes. Sólo las respuestas de categoría (frutas, muebles, flores) se consideran correctas.

Puntaje: 3 correctas=3; 2 correctas=2; 1 correcta=1; ninguna correcta=0.

### 2. Fluidez léxica (flexibilidad mental).

*“Diga todas las palabras que pueda (por ejemplo animales, plantas y objetos, pero no nombres propios ni apellidos) que comiencen con A”.* Si no responde en los primeros 5 segundos decirle *“por ejemplo, árbol”.* Si se detiene por más de 10 segundos, insista *“cualquier palabra que empiece con A”.* Tiempo: 60 segundos. Las repeticiones, derivaciones (árbol, arbolito), nombres propios y apellidos no se cuentan.

Puntaje: 10 o más palabras=3; 6 a 9=2; 3 a 5=1; menos de 3=0.

### 3. Secuencias motoras (programación).

*“Mire con atención lo que hago”;* el examinador frente al paciente realiza 3 veces la prueba de Luria (golpear con nudillo, canto y palma) con su mano izquierda. *“Con su mano derecha haga lo mismo que yo, primero juntos, después solo”.* El examinador hace la serie 3 veces con el paciente y le dice *“ahora haga lo mismo Ud. solo”.*

Puntaje: 6 series consecutivas correctas=3; 3 a 5 series correctas=2; no lo hace solo, pero sí 3 series consecutivas con el examinador=1; no logra ni siquiera imitar 3 veces=0.

### 4. Instrucciones conflictivas (sensibilidad a la interferencia).

*“Cuando yo golpeo 1 vez, debe golpear 2 veces”;* para asegurar que comprendió las instrucciones, se hace una serie de 3 ensayos: 1-1-1. *“Cuando yo golpeo 2 veces, debe golpear una”;* para asegurar que comprendió las instrucciones, se hace una serie de 2-2-2. El examinador realiza la siguiente serie: 1-1-2-1-2-2-2-1-1-2.

Puntaje: sin errores=3; 1 o 2 errores=2; más de 2 errores=1; si golpea igual que el examinador al menos 4 veces consecutivas=0.

### 5. Go-no Go (control inhibitorio).

*“Cuando yo golpeo 1 vez, debe golpear 1 vez”;* para asegurar que comprendió la instrucción, se hace una serie de 3 ensayos: 1-1-1. *“Cuando yo golpeo 2 veces, no debe golpear”;* para asegurar que comprendió la instrucción, se hace una serie de 3 ensayos: 2-2-2. El examinador realiza la siguiente serie: 1-1-2-1-2-2-2-1-1-2.

Puntaje: sin errores=3; 1 o 2 errores=2; más de 2 errores=1; golpea igual que el examinador al menos 4 veces seguidas=0.

### 6. Conducta de prehensión (autonomía del ambiente).

El examinador se sienta frente al paciente, que tiene las manos sobre sus rodillas, con las palmas hacia arriba. El examinador acerca lentamente sus manos hasta tocar las del paciente para ver si se las toma espontáneamente. Si lo hace, dice *“ahora, no me tome las manos”* y vuelve a tocárselas.

Puntaje: no le toma las manos=3; duda o pregunta qué tiene que hacer=2; las toma sin vacilar=1; las toma aún después de decirle que no lo haga=0.

Puntaje:

1. Semejanzas=3 −2 −1 −0
2. Fluencia lexical=3 −2 −1 −0
3. Secuencias motoras=3 −2 −1 −0
4. Instrucciones conflictivas=3 −2 −1 −0
5. Go-no go=3 −2 −1 −0
6. Conducta de prehension=3 −2 −1 −0

Total= /18
